# Gut microbiota of the young ameliorates physical fitness of the aged in mice

**DOI:** 10.1186/s40168-022-01386-w

**Published:** 2022-12-26

**Authors:** Kwang H. Kim, Yusook Chung, Ji-Won Huh, Dong Jin Park, Yejin Cho, Yeseul Oh, Haengdueng Jeong, Jaekyung Yoon, Ju-Hee Kang, Hae-Sol Shin, Hyoung-Chin Kim, Soon-Kyeong Kwon, Kyoung Yul Seo, Seung Hyun Oh, Je Kyung Seong, Sang-Jun Ha, Ki Taek Nam, Jihyun F. Kim

**Affiliations:** 1 Severance Biomedical Science Institute, Brain Korea 21 Project for Medical Science, Yonsei University College of Medicine, Seoul, Korea; 2 Department of Systems Biology, Division of Life Sciences, and Institute for Life Science and Biotechnology, Yonsei University, Seoul, Korea; 3 Department of Biochemistry and Division of Life Sciences, Yonsei University, Seoul, Korea; 4 College of Pharmacy, Gachon University, Incheon, Korea; 5 Korea Mouse Sensory Phenotyping Center (KMSPC), Yonsei University College of Medicine, Seoul, Korea; 6 Laboratory Animal Resource Center, Division of Bioinfrastructure, Korea Research Institute of Bioscience and Biotechnology (KRIBB), Daejeon, Korea; 7 Division of Applied Life Science (Brain Korea 21), Gyeongsang National University, Jinju, Korea; 8 Korea Mouse Phenotyping Center (KMPC), Seoul National University, Seoul, Korea; 9 Microbiome Initiative and Strategic Initiative for Microbiomes in Agriculture and Food (iMAF), Yonsei University, Seoul, Korea

**Keywords:** Programmed aging, C57BL/6, Microbiome, Sarcopenia, Ki-67

## Abstract

**Background:**

Aging is a natural process that an organism gradually loses its physical fitness and functionality. Great efforts have been made to understand and intervene in this deteriorating process. The gut microbiota affects host physiology, and dysbiosis of the microbial community often underlies the pathogenesis of host disorders. The commensal microbiota also changes with aging; however, the interplay between the microbiota and host aging remains largely unexplored. Here, we systematically examined the ameliorating effects of the gut microbiota derived from the young on the physiology and phenotypes of the aged.

**Results:**

As the fecal microbiota was transplanted from young mice at 5 weeks after birth into 12-month-old ones, the thickness of the muscle fiber and grip strength were increased, and the water retention ability of the skin was enhanced with thickened stratum corneum. Muscle thickness was also marginally increased in 25-month-old mice after transferring the gut microbiota from the young. Bacteria enriched in 12-month-old mice that received the young-derived microbiota significantly correlated with the improved host fitness and altered gene expression. In the dermis of these mice, transcription of *Dbn1* was most upregulated and DBN1-expressing cells increased twice. *Dbn1*-heterozygous mice exhibited impaired skin barrier function and hydration.

**Conclusions:**

We revealed that the young-derived gut microbiota rejuvenates the physical fitness of the aged by altering the microbial composition of the gut and gene expression in muscle and skin. *Dbn1*, for the first time, was found to be induced by the young microbiota and to modulate skin hydration. Our results provide solid evidence that the gut microbiota from the young improves the vitality of the aged.

Video Abstract

**Supplementary Information:**

The online version contains supplementary material available at 10.1186/s40168-022-01386-w.

## Background

Aging is an inevitable deterioration of a structure and integrity of an organism, accompanying the functional decays in every aspect of physiology [1–4]. Understanding and regulating the aging process have been of great interest throughout the history [5, 6]. Manifestations of senescence occur not only at the cellular level such as shortened telomere and cell cycle arrest, but at the organ and organism levels—impaired cognitive function, dry eye, chronic gut inflammation, decreased skeletal muscle mass, skin wrinkling, *et cetera* [1].

Trillions of microorganisms reside in the mucosal surface of the host intestine, where they dynamically interact with a myriad of factors such as foods, host-derived molecules, and other microbes [7, 8]. The gut microbiota is considered a virtual organ of the host that plays a key role in maintaining immune and metabolic homeostasis [9, 10]; dysbiosis—disruption of microbiota homeostasis—has been repeatedly implicated in various diseases including inflammatory bowel disease, obesity, metabolic liver diseases, type 2 diabetes, cardiovascular diseases, cancers, and even neurological disorders [10, 11]. Accumulating evidence suggests that the gut microbiota changes with host aging [12]. Moreover, the gut microbiota is emerging as a key player underlying several aging-related disorders [13, 14].

Recently, several studies have been conducted to demonstrate the causality of the gut microbiota on host health by transferring them to recipient groups of different ages. Some of them found that the gut microbiota derived from the aged promoted inflammation and in two cases cognitive decline as well [15–17]. Another study showed that the old-derived microbiota conferred obesogenic characteristics to the young germ-free mice [18]. In the opposite direction, the gut microbiota from young African turquoise killifish was, for the first time in vertebrates, revealed to extend the life span of old killifish with improved muscle function and locomotor activity [19]. Transcriptome analysis also indicated that gut aging is associated with increased inflammation and reduced proliferation. The killifish is a good model for aging-related studies of vertebrates as it only lives a few months. However, not only it is distantly related to mammals such as humans, but its gut microbiota differs from that of terrestrial vertebrates [20–22]. Further, underlying mechanisms for the counteracting effects of the young-derived microbiota on physical fitness including muscle and skin properties have yet to be explored in higher vertebrates.

The aim of this study is to systematically determine the revitalizing effects of the gut microbiota derived from the young on the physiology of the aged and to investigate the microbial and host genetic factors that play pivotal roles in the process. Thus, we transplanted the fecal microbiota from young (5 weeks after birth) mice to old (12 months) and very old (25 months) ones. We then monitored the gut microbiota profiles and examined the organ mass, serology, immune cellularity, gene expression profile, and aging manifestations in 2 months. We further analyzed microbial taxa that correlate with changes in the phenotypes and gene expression in mice. Mice heterozygous for *Dbn1*, which was the most upregulated gene in old mice after receiving the young-derived gut microbiota, were examined for selected phenotypes.

## Methods

### Mice

Animal experiments were approved by the Institutional Animal Care and Use Committee of Gachon University (IACUC number: GIACUC-R2019030) and the Institutional Animal Care and Use Committee of Yonsei University (IACUC number: 2018-0145), and the experiments were compliant with the Guide for the Care and Use of Laboratory Animals.

Reaching puberty, mammalian females are considered sexually mature after menarche, which occurs as early as 4 weeks after birth in mice [23, 24]. Reproductively senescent perimenopause females (between 9 and 12 months in mice) undergo a drastic decrease in sex hormone levels, which significantly contributes to the initiation of programmed aging [25–27]; menopause marks the final and irreversible termination of the female reproductive life span [28]. Survivorship drops off markedly for mice over 24 months of age and can be considered very old [29]. Based on these dispositions, female mice at 5 weeks were defined as young and two aged ones at 12 and 25 months as old and very old, respectively, in this study.

In late July 2019, C57BL6 female mice at 3 weeks, 12 months, and 25 months were obtained from the Laboratory Animal Resource Center, Daejeon, Korea, maintained in a specific pathogen-free (SPF) facility under a 12-h light/dark cycle. Mice were acclimated to the laboratory animal facility at Gachon University for 2 weeks before starting the experiment. Mice were fed with a normal Chow diet. Each individual mouse was tail-tagged for identification and randomly assigned to the treatment groups, which allows for matching different types of data including microbial structure, RNA sequencing, and aging-associated phenotypes.

*Dbn1*^*Het*^ C57BL/6 mice (16-week-old) used for validation of *Dbn1* for its involvement in skin properties were available from Korea Mouse Phenotyping Center (KMPC). *Dbn1*^*WT*^ and *Dbn1*^*Het*^ mice were maintained by crossbreeding *Dbn1*^*Het*^; complete loss of the *Dbn1* gene (*Dbn1*^-/-^) resulted in embryonic lethality. Four weeks after birth, offspring were separated from the cage housing the mother and genotyped using the following primers: 5′–GTCCTTCCTCTTGGTCATTCCC and 5′–TGGAGAAACCAGGGAGATGTTG (for wild type), 5′–GCTACCATTACCAGTTGGTCTGGTGTC and 5′–CAGAGCCCAAGACTAATACACCC (for knockout).

### Fecal microbiota transplantation

To prepare donor samples for fecal microbiota transplantation (FMT), we collected fresh feces after 2 weeks of acclimation by gently rubbing the belly from young (Y; 5 weeks: adolescence), old (O; 12 months: perimenopause), and very old (VO; 25 months: senescence) C57BL6 female mice, respectively, and maintained them under the strict anaerobic condition throughout the process. The feces were pooled by groups and mixed with PBS supplemented with 10% glycerol (30 mg feces/mL PBS; ca. 5 × 10^8^ colony-forming units/mL) and thoroughly homogenized via vortexing. After sedimentation for 10 min, the supernatant was filtered through a 40-μm cell strainer and the homogenous aliquots were stored in a refrigerator at −80 °C.

Approximately 100 μL of the microbial suspension was transferred into each C57BL6 female mouse by oral gavage twice a week for a total of 8 weeks using autoclaved feeding needles. Superscript was used to describe the FMT donor. Thus, if feces from the young mice (Y) are given to 12-month-old or 25-month-old groups, they are designated as Old^Y^ or vOld^Y^, respectively. As a control (C), 100 μL of PBS with 10% glycerol was administrated to old (Old^C^) or very old (vOld^C^) mice. In the same manner, feces from old or very old mice (O or VO) were respectively given to 12- or 25-month-old groups, resulting in Old^O^ or vOld^VO^ mice. All the recipient mice were housed under the SPF condition and weighed twice a week during the FMT timeline. Examination of passive avoidance, grip strength, skin properties, and visual abilities was performed 3 days after the last FMT. Then, all mice were euthanized and sacrificed for the measurement of physiological changes.

To generate microbiota-depleted old (xOld) mice by clearing out pre-existing gut microbiota, drinking water containing antibiotics (1 g/L ampicillin, 0.5 g/L vancomycin, 1 g/L metronidazole, 1 g/L neomycin, and 2.5 mg/L amphotericin B) was given to mice ad libitum during 7 days and withdrawn 24 h before FMT. When we tried the bacterial clearance in very old mice to make xvOld, they failed to tolerate the antibiotic treatment and died.

### Fecal microbiota analysis

DNA was extracted using a Fast DNA SPIN kit for fecal samples (MP Biomedicals, Irvine, CA, USA), according to the manufacturer’s instructions. The V3-V4 region of the 16S ribosomal RNA (rRNA) gene was sequenced on an Illumina MiSeq platform by Chun Laboratory (Seoul, Korea) using sequence-specific primers (337F: CCTACGGGA(N)GGCWGCAG, 806R: GACTACHVGGGTM(A)TCTAAT). Raw sequencing data were analyzed using the QIIME2 pipeline (version 2017.10). The q2-demux plugin with DADA was used to de-multiplex and trim the adaptor sequences, and sequence variants were clustered into amplicon sequence variants (ASVs). The phylogenetic tree was constructed using q2-alignment and q2-phylogeny plugins. Sequence variants were taxonomically assigned using the SILVA reference database [30]. The feature table and phylogenetic distances were imported into R for downstream analysis.

Alpha diversity indices including the number of observed ASVs, Chao1, and Shannon’s diversity were calculated using phyloseq and vegan R packages. Linear discriminant analysis effect size (LEfSe) was used to identify microbial biomarkers using the factorial Kruskal-Wallis test (*P* < 0.05) [31]; the threshold of logarithmic LDA score was 2.0. Spearman’s correlation coefficient was calculated for bacterial species that have more than 0.5% relative abundance in 12-month-old recipient mice using the psych R package.

### Passive avoidance test

To examine the cognitive response of mice, a passive avoidance test was performed. A mouse was placed in a light chamber for 30 s and allowed to move to a dark chamber upon gate opening. The gate was immediately closed after the mouse moved to the dark chamber, and an electric foot shock (0.5 mA) was administered for 2 s. The time between the gate opening and entering of the mouse into the dark chamber was recorded as a training time. Then, the mouse was returned to the home cage. After 24 h, the mouse was returned to the light chamber for 30 s, and then, the gate of the dark chamber was opened. The time for the mouse to enter the dark chamber after the gate opening was recorded as a retention time.

### Measurement of grip strength

Forelimb grip strength test was performed using GSM Grip-Strength Meter (Ugo Basile, 47200). The mouse was placed on an acrylic panel and the peak grip force of the mouse on the metal wire of the apparatus was measured in grams. The peak force was averaged from three independent trials.

### Measurement of skin properties

The day before measuring skin hydration phenotypes, mouse dorsal hair was carefully shaved. Skin hydration was measured using MoistureMeterSC (Delfin) which registers a probe to the skin surface until skin hydration is stabilized. The capacitance is expressed in arbitrary units (AU). Transepidermal water loss (TEWL) was measured using VapoMeter (Delfin) that registers a probe to the skin surface until TEWL is stabilized (less than 1 min). The result is expressed in g/hm^2^.

### Visual measurement

Spatial frequency thresholds (i.e., visual acuity) were assessed by optokinetic nystagmus (OKN) using a virtual optokinetic system (OptoMotry, Cerebral Mechanics, Medicine Hat, Alberta, Canada). A video camera at the ceiling of the device records and transfers images to the connected computer. The clockwise movement of the drift grid tracked the movement of the left eye, and the counterclockwise movement tracked the reaction of the right eye of the mouse. The experimenter judged whether the head and body of the mouse were tracking the direction of the drift grid rotation. If tracking was unclear or absent, the process was repeated. The maximum spatial frequency capable of driving head tracking was determined.

For the intraocular pressure (IOP) test, mice were anesthetized using an intraperitoneal injection of xylazine (10 mg/kg; Rompun®, Bayer Animal Health) and zolazepam and tiletamine (30 mg/kg; Zoletil 50®, Virbac, Carros, France). IOP was measured using a rebound tonometer (Icare® TONOLAB tonometer, Colonial Medical Supply, Franconia, NH, USA), according to the manufacturer’s instructions. One trial result was obtained after six consecutive measurements, and the mean of consecutive trials was used for analyses.

Electroretinogram (ERG) analysis was performed using Micron Ganzfeld ERG (Phoenix Research Labs, Pleasanton, USA). The mouse was dark-adapted at least 12 h before the experiment for scotopic testing (rod cell response). After anesthesia, the pupil dilated as previously described. Once the pupil was fully dilated, we applied hypromellose 2.5% (Goniovisc®) and inserted the electrodes. ERG was recorded using Micron Ganzfeld ERG, according to the manufacturer’s instructions. Scotopic ERG was obtained with increasing flash intensity at a range of −1.7 log cd/s/m^2^ to 1.9 log cd/s/m^2^. Mice were light-adapted for 15 min prior to the cone cell response experiment. Photopic ERG was performed with increasing flash intensity at a range of −0.5 log cd/s/m^2^ to 4.1 log cd/s/m^2^. Values were based on the average of 10 responses to light stimuli. The implicit times of rod and cone cell response were determined.

### Blood chemistry

For blood sample collection, the three groups of FMT recipients were euthanized using CO_2_ gas, and blood was directly obtained from the heart using 26G 1 mL syringe. To isolate serum from the total blood, blood samples were placed in the MiniCollect® tube (Greiner Bio-One, Kremsmünster, Austria) and centrifuged at 1 × 10^4^ rpm for 5 min at 25 °C. The levels of aspartate aminotransferase (GOT), alanine aminotransferase (GPT), creatinine phosphokinase (CPK), alkaline phosphatase (ALP), blood urea nitrogen (BUN), amylase (AMYL), creatinine (CRE), albumin (ALB), triglyceride (TG), total bilirubin (TBIL), total cholesterol (TCHO), r-glutamyltransferase (GGT), lactate dehydrogenase (LDH), high-density lipoprotein-cholesterol (HDLC), and creatine kinase-mb (CKMB) in the serum samples (10 μL each) were measured using DRI-chem 4000i (Fuji, Minato, Japan) automated clinical chemistry analyzer.

### H&E staining

The mouse tissues were fixed with ice-cold 4% paraformaldehyde and embedded in paraffin. The tissues were cut into 5-μm thick sections; deparaffinized with xylene three times for 20 min each, 100% EtOH three times for 10 min each, 90% EtOH twice for 10 min each, and 75% EtOH for 10 min; and stained with hematoxylin and eosin (H&E). Slides containing the stained tissue were dehydrated and mounted on Shandon Synthetic Mount (Thermo, Inc., Waltham, MA, USA). The diameter of the myofiber in the hindlimb skeletal muscle was determined using digital images of the sections using the ToupView program (http://www.touptek.com).

### Immunohistochemical and immunofluorescence staining

For immunohistochemistry, the tissues were fixed with ice-cold 4% paraformaldehyde in PBS and mounted on paraffin blocks. Samples were cut into 3 μm sections, deparaffinized, and rehydrated in PBS. Antigens were then retrieved for 15 min at high pressure in Target Retrieval Solution (Dako, Santa Clara, CA, USA). Then, the specimens were chilled on ice for 1 h, washed with PBS three times for 5 min each and blocked with 3% H_2_O_2_ in PBS for 30 min to eliminate the endogenous peroxidase. The slides were washed with PBS, blocked for 2 h at 25 °C with Serum-Free Protein Block (Dako), probed at 4 °C overnight with the primary antibodies (1/1000 dilution), stained for 30 min with horseradish peroxidase-conjugated anti-mouse (Dako) or anti-rabbit IgG (Dako) secondary antibody, and developed with Liquid DAB+ Substrate Chromogen System (Dako). Finally, the specimens were counterstained with Mayer’s hematoxylin (Dako) and mounted on Shandon Synthetic Mount (Thermo).

For immunofluorescence, tissue sample preparation and blocking were performed in the same manner as described in immunohistochemistry. Then, the slides were probed at 4 °C overnight with the primary antibodies (1/1000 dilution), labeled for 2 h with Cy3-conjugated goat anti-rabbit IgG (Thermo) or A488 donkey anti-mouse IgG (Thermo), and stained with DAPI (Sigma, Burlington, MA, USA) for 15 min. Finally, the slides were mounted on ProLong Gold antifade reagent (Thermo), imaged on an Axio Imager M2 fluorescence microscope (Zeiss, Oberkochen, Land Baden-Württemberg, Germany), and analyzed using Zeiss Zen Blue Edition. The following primary antibodies were used for staining: anti-Ki67 (Abcam, Cambridge, UK), anti-DBN1 (Novus, Denver, CO, USA), anti-ITGB4 (Abcam), and anti-KRT10 (Abcam).

### Multiplex cytokine and chemokine bead array assays

Multiplex bead array assays for serum cytokine quantification were performed using (#740446; BioLegend, San Diego, CA, USA) according to the manufacturer’s instructions. Serum samples were analyzed via a CytoFLEX platform (Beckman Coulter, Brea, CA, USA).

### Immune cell preparation

Single-cell suspensions of the spleen, lymph nodes, and liver were prepared by mechanical homogenization of the organs and passing through 40-μm or 70-μm cell strainer (BD Biosciences, San Jose, CA, USA). For the spleen, erythrocytes were removed using ACK lysing buffer (Thermo). Liver cells were purified by density gradient centrifugation on a 67% and 44% Percoll gradient (GE Healthcare, Chicago, IL, USA).

### Flow cytometry

Cells were seeded in a 96-well plate and stained with fluorochrome-conjugated antibodies for 20 min at 4 °C in the dark. Cells were subsequently washed with PBS containing 2% fetal bovine serum. The following antibodies were used: CD8 (53-6.7; BD Biosciences), CD62L (MEL-14; BD Biosciences), CCR7 (4B12; eBioscience, San Diego, CA, USA), CD103 (2E7; eBioscience), CD25 (PC61; BioLegend), CD45.2 (104; BioLegend), CD44 (IM7; BioLegend), CD69 (H1.2F3; BioLegend), and CD4 (RM4-5; BioLegend). Cells were stained with LIVE/DEAD® Fixable Blue Dead Cell Stain (Invitrogen, Carlsbad, CA, USA) to discriminate dead cells. All stained samples were acquired using CytoFLEX LX instrument (Beckman Coulter), and the data were analyzed using the FlowJo software (BD Biosciences).

### RNA sequencing

Total RNA was isolated from the distal colon, quadriceps femoris muscle (quad muscle), and back skin, respectively, using Trizol reagent (Invitrogen). RNA quality was assessed by Agilent 2100 bioanalyzer using the RNA 6000 Nano Chip (Agilent Technologies, Amstelveen, The Netherlands), and RNA quantification was performed using ND-2000 Spectrophotometer (Thermo). For messenger RNA sequencing, a complementary DNA library was constructed using QuantSeq 3’ mRNA-Seq Library Prep Kit (Lexogen, Inc., Austria), according to the manufacturer’s instructions. Briefly, 500 ng of the total RNA was prepared and an oligo-dT primer containing an Illumina-compatible sequence at its 5′ end was hybridized to the RNA and reverse transcribed. After degradation of the RNA template, second strand synthesis was initiated using a random primer containing an Illumina-compatible linker sequence at its 5′ end. The double-stranded DNA library was purified using magnetic beads to remove all reaction components. The library was amplified to obtain the complete adapter sequences required for cluster generation. The finished library was purified to remove PCR components. High-throughput sequencing was performed using single-end 75-bp reads using NextSeq 500 (Illumina, Inc., USA).

QuantSeq 3′ mRNA-Seq reads were aligned using Bowtie2. Bowtie2 indices were either generated from the genome assembly sequence or representative transcript sequences for aligning to the genome and transcriptome. The alignment file was used for assembling transcripts, estimating their abundances, and detecting differential expression of genes. Raw read counts were normalized and differentially expressed genes (DEGs) were identified using the edgeR package (*P* < 0.01). The DEGs were determined based on read counts from unique and multiple alignments using coverage in Bedtools. Functional annotation of DEGs was performed using DAVID (http://david.abcc.ncifcrf.gov/) and MEDLINE (http://www.ncbi.nlm.nih.gov/) databases.

### Correlation analysis

Complete hierarchical clustering based on Euclidean distance was performed to classify co-occurred species into clusters using the hclust function in the R package, and four clusters were obtained by cutting off the dendrogram tree at height 6. Spearman’s correlation coefficient was calculated to examine the relationship between DEGs and microbial composition. *P* values were corrected for multiple comparison problems using Benjamini-Hochberg method with a 0.05 cutoff.

### Statistical analysis

Statistical analyses of host phenotype data were performed using Prism software (GraphPad). Raw data were subjected to one-way ANOVA to evaluate statistical significance between at least three groups, and pairwise comparison was conducted using Student’s *t* test. Means were considered significantly different at *P* < 0.05. Statistical details, including sample sizes for each experiment, are provided in the relevant figure legends.

## Results

### Microbiota of the young improves the muscle and skin properties of the aged

**Fig. 1:**
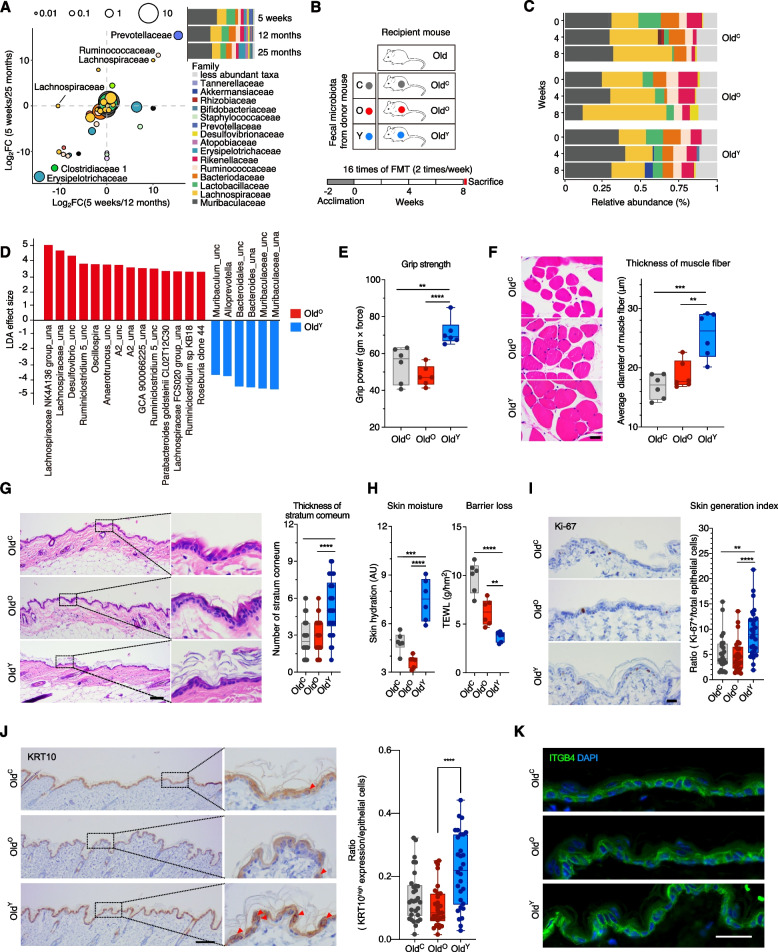
Fecal microbiota transplantation of young mice invigorates the fitness of old mice. **A** Relative abundance of gut microbiota at the family level in mice at 5 weeks (young, adolescence), 12 months (old, perimenopause), and 25 months (very old, senescence) of age. Scatter plot of the fold changes in the relative abundances of each species between the groups: 5 weeks against 12 months and 5 weeks against 25 months. Dot size indicates the mean proportion of each species. **B** Schematic illustration of fecal microbiota transplantation (FMT) to 12-month-old mice (Old). The matrix describes three donor groups: C, PBS with 10% glycerol for control; O, old at 12 months; Y, young at 5 weeks. The recipient mice are designated Old^C^ (*n* = 6), Old^O^ (*n* = 6) and Old^Y^ (*n* = 6), respectively. After 2 weeks of the acclimation period, FMT was performed 16 times for 8 weeks. Mice phenotypes were measured 3 days after the last FMT. **C** Relative abundance of the gut microbiota in Old during FMT. Samples were grouped by the fecal donor and ordered by collection time. **D** Linear discriminant analysis effect size of samples between Old^O^ and Old^Y^ at week 8. _una, unassigned; _unc, uncultured. **E** Grip strength of contralateral forelimb for Old^C^, Old^O^, and Old^Y^. **F** Hematoxylin and eosin (H&E) stain images of the hindlimb skeletal muscle fiber and average diameter of the muscle fiber in each group. Scale bar = 20 μm. **G** H&E stain images of the epidermal skin (left) and the average number of stratum corneum layers (right). Scale bar = 100 μm. **H** Skin hydration (left) and TEWL (right). **I**, **J** Immunohistochemical staining and quantification of Ki-67 (**I**) and KRT10 (**J**) in the epidermal skin layer. Scale bar = 100 μm. **K** Immunofluorescence staining of ITGB4. Scale bar = 25 μm. Box and whiskers. Student’s *t* test. *P < 0.05, **P < 0.01, ***P < 0.001, ****P < 0.0001

To investigate whether the phenotypes of aged mice could be improved by the gut microbiota of the young, we first designated three different age groups of female C57BL/6 mice and compared the baseline microbiota via rRNA gene sequencing. The three groups from which fresh fecal samples were collected are as follows: 5 weeks after birth for young (represents adolescence), 12 months for old (perimenopause), and 25 months for very old (senescence). Differentially abundant microbes included *Prevotellaceae* (belongs to *Bacteroidetes*) and *Ruminococcaceae* (*Oscillospiraceae*; *Firmicutes*) in the young mice and *Erysipelotrichaceae* (*Firmicutes*) and *Clostridaceae* 1 (*Firmicutes*) in the two old groups; however, the abundance of *Muribaculaceae* (*Bacteroidetes*) was comparable between the groups (Fig. 1A).

Fecal contents were repeatedly transferred to mice at 12-month-old mice (Old) by oral gavage for 8 weeks (twice a week) to ensure the establishment of donor microbiota: vehicle control (C), 5 weeks (Y), and 12 months (O) (Fig. 1B). The fecal microbiota of the recipient mice was longitudinally collected at weeks 0, 4, and 8. The abundance of bacterial families including *Muribaculaceae*, *Lachnospiraceae*, *Lactobacillaceae*, *Bacteroidaceae*, *Ruminococcaceae*, and *Rikenellaceae* differently changed over time depending on the source of microbiota (Fig. 1C). To screen for the microbial biomarkers that are specific for each group, LEfSe was applied to the 8-week microbiota data. Results showed that species belonging to *Muribaculaceae*, *Bacteroidaceae*, and *Prevotellaceae* are most discriminative in old mice that received microbiota derived from the young (Old^Y^), whereas species of *Lachnospiraceae*, *Desulfovibrionaceae*, *Ruminococcaceae*, and *Tannerellaceae* were discriminative in old mice that received microbiota from the old (Old^O^) (LDA effect size ≥ 2.0; Fig. 1D). There was no significant difference in observed ASVs, Chao1, and Shannon’s diversity index across the recipient groups (Fig. S1A).

During the experiment, the recipient mice appeared healthy and maintained their body and organ weights, implying that the repeated oral gavages were not excessively stressful for the recipients (Fig. S1B, C). Data from the blood chemistry analysis showed that FMT did not change the levels of most systemic metabolic indicators except a slight increase in amylase level in Old^Y^ (Fig. S1D). In addition, the exogenous microbiota did not alter two well-known aging-associated phenotypes, which are avoidance-learning memory and visual abilities such as visual acuity, IOP, and response time of rod and cone cells (Fig. S1E, F). However, when we performed forelimb grip strength test that is a common method to measure neuromuscular function of rodents, Old^Y^ mice had 30–50% stronger grip strength than Old^C^ and Old^O^ (*P*_YvsO_ = 0.000079; Fig. 1E). In line with the result, the diameter of the muscle fiber was significantly increased in Old^Y^, suggesting the improved muscle function by the young-derived microbiota (*P*_YvsO_ = 0.0042; Fig. 1F). In skin, the number of stratum corneum layer (outermost barrier of the epidermis), was highly increased in Old^Y^ (5.3±2.23 in Old^Y^, 2.83±1.36 in Old^O^; Fig. 1G). Consequently, the ability to retain moisture was dramatically enhanced as shown in the skin hydration assessment (7.47±1.29 AU in Old^Y^, 3.66±0.38 AU in Old^O^) and TEWL measurement (3.71±0.52 g/hm^2^ in Old^Y^, 6.24±1.34 g/hm^2^ in Old^O^; Fig. 1H). The epithelium of Old^Y^ had more Ki-67-positive proliferating cells, and in situ expression of keratin 10 (KRT10) was also elevated in Old^Y^ compared to Old^O^, but integrin beta 4 (ITGB4) was comparable (Fig. 1I–K).

To investigate the effects of the young-derived microbiota on the very old mice, we also performed young-to-old microbiota transfer with 25-month-old mice (vOld) using three donor groups: vehicle control, 5 weeks (Y), and 25 months (VO) (Fig. S2A). During 0, 4, and 8 weeks, most of the microbial families that are abundant in Old were present in vOld as well, with the exception of *Bifidobacteriaceae* in vOld (Fig. S2B). LEfSe indicated that biomarkers belonging to *Prevotellaceae* and *Muribaculaceae* that were retrieved in Old^Y^ were replicated in vOld^Y^ at week 8, as well as *Roseburia* (*Lachnospiraceae*) in vOld^VO^, but a species of *Eubacteriaceae* was newly represented in vOld^VO^ (Fig. S2C). There was no significant difference in the number of observed ASVs, Chao1, and Shannon’s diversity index across the recipients (Fig. S2D). No significant changes were observed in body weight, organ mass, and the number of stratum corneum layers; only the relative brain weight (*P*_YvsVO_ = 0.004) and the thickness of muscle fiber (*P*_YvsVO_ = 0.085) were increased in vOld^Y^ (Fig. S2E–H).

**Fig. 2:**
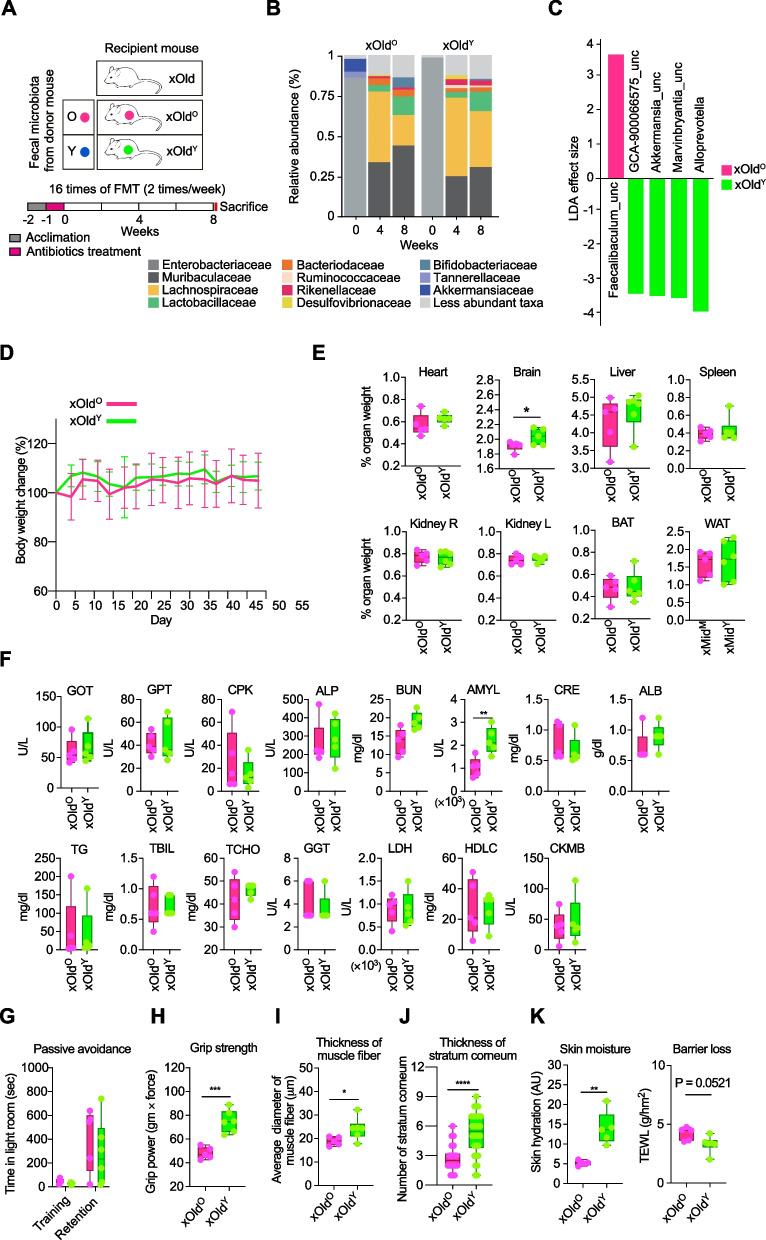
Physiological characteristics of Abx-treated old mice. **A** Schematic illustration of FMT to antibiotics-treated 12-month-old mice (xOld). The matrix describes two donor groups: O, old at 12 months; Y, young at 5 weeks. The recipient mice were designated as xOld^O^ (*n* = 5) and xOld^Y^ (*n* = 6). After a week of acclimation followed by a week of antibiotic treatment, FMT was repeated 16 times for 8 weeks. **B** Relative abundance of the gut microbiota in xOld during FMT. Samples were grouped by the fecal donor and ordered by collection time. **C** Linear discriminant analysis effect size of samples between xOld^O^ and xOld^Y^ at week 8. _una, unassigned; _unc, uncultured. **D** Body weight changes in xOld during FMT. Mice were housed in a SPF facility and weighed twice a week during FMT treatment. Results are represented as mean ± SD. **E** Relative organ weight in xOld after FMT. Each group of mice was euthanized after FMT and the heart, brain, liver, spleen, kidney, BAT, and WAT were weighed. **F** Blood chemistry analysis of serum from the treatment groups. **G** Passive avoidance test. Total time in the illuminated chamber was recorded as training time on day 1 and retention time was recorded on day 2. **H** Grip strength of contralateral forelimb. **I** Average diameter of muscle fiber. **J** Average number of stratum corneum layers. **K** Skin hydration (left) and TEWL (right) values. Box and whiskers. Student’s *t* test. *P < 0.05, ** *P* < 0.01, *** *P* < 0.001, and **** *P* < 0.0001

Further, we designed an experiment in which mice were pre-treated with an antibiotic cocktail (Abx) before FMT to remove the resident gut microbiota and to exclude the involvement of pre-existing microbes (Fig. 2A). By 4 weeks after the first FMT to Abx-treated 12-month-old mice (xOld), the taxonomic structure was restored harboring high proportion of *Muribaculaceae*, *Lachnospiraceae*, *Lactobacillaceae*, and *Bacteroidaceae*, which was similar to those of the gut microbiome of SPF mice described above and the common murine gut microbiome (Fig. 2B)*.* Consistent with the results of SPF mice, *Alloprevotella* (*Prevotellaceae*) was found to be most discriminative in Abx-treated old mice that received microbiota from the young (xOld^Y^), and *Akkermansia* that is predominant in microbiota derived from the young was also observed (Fig. 2C). Body weight of the recipients was stable during FMT, and most of the phenotypic changes were reproduced without pre-existing microbiota. There were no significant differences in organ weights except brain in xOld^Y^ mice (2.04±0.11 % organ weight in xOld^Y^, 1.91±0.07 % in xOld^O^), and the levels of metabolic indicators except amylase and aversive memory were not changed (Fig. 2D–G). Most importantly, muscle fitness and skin properties were consistently improved (Fig. 2H–K). Very old (25 months) mice that also received the same dose of Abx for microbiota clearance failed to tolerate the toxicity of Abx and most died; results from xvOld were excluded in further analysis.

Although FMT has been reportedly implicated in the intestinal immune responses [32], our study showed that there was no significant alteration in the serum cytokine profile or T cell population in the spleen, liver, and mesenteric or inguinal lymph nodes (Fig. S3). These results highlight that transplanting the young-derived microbiota can rejuvenate the aging-associated phenotypes of the skin and muscle without eliciting severe inflammation.

### Young microbiota alters host gene expression in the colon, muscle, and skin

**Fig. 3:**
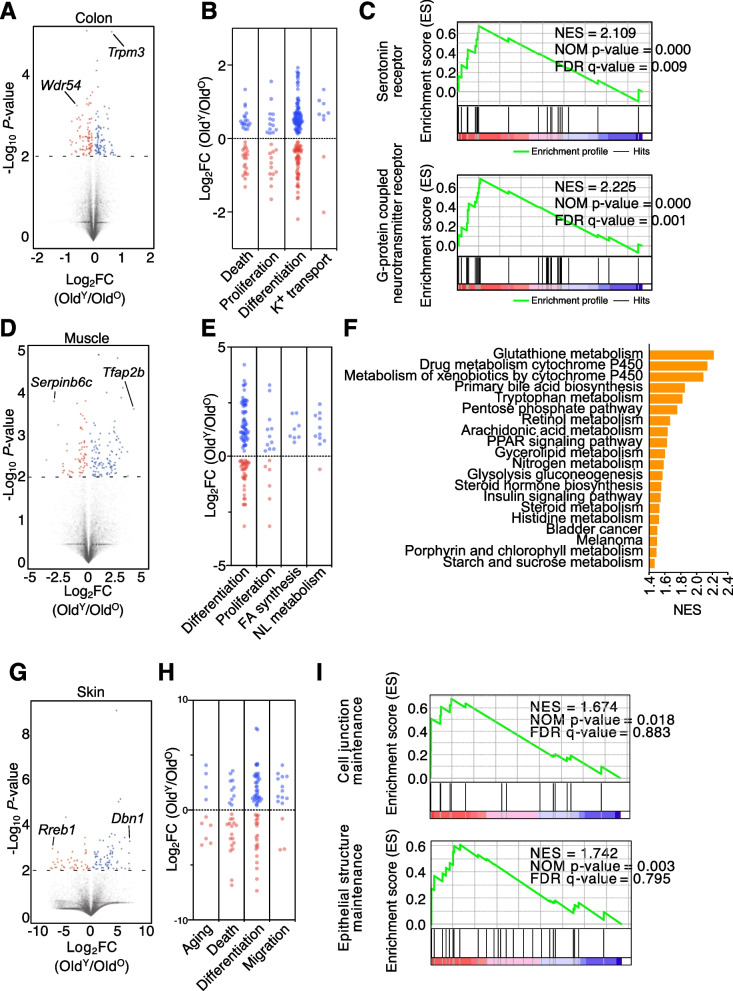
Genes in old mice are upregulated by FMT from young mice. **A** Volcano plot of fold changes (FCs) and *P* values for Old^Y^ versus Old^O^ in the colon (*n* = 3). Gene (*P* ≤ 0.001) names with the highest fold change in Old^O^ or Old^Y^ are shown. **B** FC levels of death-related (39), proliferation-related (26), differentiation-related (142), and potassium transport-related (9) marker genes in colon. **C** Gene set enrichment analysis (GSEA) for Old^Y^ vs. Old^O^ in the colon. GSEA reveals positive enrichment of serotonin receptor activity and G-protein-coupled neurotransmitter receptor activity gene sets in the Old^Y^ colon. **D** Volcano plot of FCs and *P* values for Old^Y^ vs. Old^O^ in the muscle (*n* = 3). **E** FC levels of differentiation-related (100), proliferation-related (17), fatty acid synthesis-related (8), and neutral lipid metabolism-related (11) marker genes in the muscle. **F** Top 20 KEGG pathways overrepresented in the muscle of Old^Y^ (*n* = 3) based on the NES score. **G** Volcano plot of FCs and *P* value for Old^Y^ (*n* = 2) vs. Old^O^ (*n* = 3) in skin. **H** FC levels of aging-related (10), death-related (27), differentiation-related (58), and migration-related (15) marker genes in skin. **I** GSEA for Old^Y^ vs. Old^O^ in skin. GSEA reveals enrichment of “cell junction maintenance” and “epithelial structure maintenance” in the Old^Y^ skin. *P* value cutoff for DEGs is 0.01

To characterize the changes in host gene expression after FMT, we performed RNA sequencing (RNA-seq) of three tissue homogenates of the colon, quad muscle, and back skin samples at week 8. Analysis of the RNA-seq data from the colon showed 89 and 91 genes expressed more in either Old^Y^ or Old^O^, respectively (Fig. 3A). More genes annotated to be involved in cell differentiation or K^+^ ion transport were upregulated in Old^Y^, and more death-associated genes were expressed higher in Old^O^ (Fig. 3B and Table S1). Furthermore, gene set enrichment analysis (GSEA) based on the KEGG database showed that receptor-signaling pathways including those related to a serotonin receptor and a G-protein-coupled neurotransmitter receptor had more genes significantly upregulated in Old^Y^ than Old^O^; however, no inflammation-related signaling pathways were detected (Fig. 3C).

In the muscle, we found that 62 and 109 genes, including *Tfap2b* and *Serpinb6c*, respectively, were expressed more in either Old^Y^ or Old^O^ (*P* < 0.05; Fig. 3D). More genes involved in cell differentiation, proliferation, fatty acid synthesis, or neutral lipid metabolism pathways showed higher expression in Old^Y^ (Fig. 3E and Table S2), and glutathione (the most abundant intracellular anti-oxidant thiol) metabolism was overrepresented in Old^Y^ according to GSEA (Fig. 3F). In skin, 47 and 73 genes, including *Dbn1* and *Serpinb6c*, respectively, were expressed more in Old^Y^ or Old^O^ (Fig. 3G). While more aging- or death-associated genes were expressed higher in Old^O^, more genes involved in cell differentiation and migration were highly expressed in Old^Y^ (Fig. 3H and Table S3). In addition, GSEA showed that more genes related to cell junction maintenance and epithelial structure maintenance were expressed in Old^Y^ than in Old^O^ (Fig. 3I).

### Certain microbial clusters link to rejuvenation

**Fig. 4:**
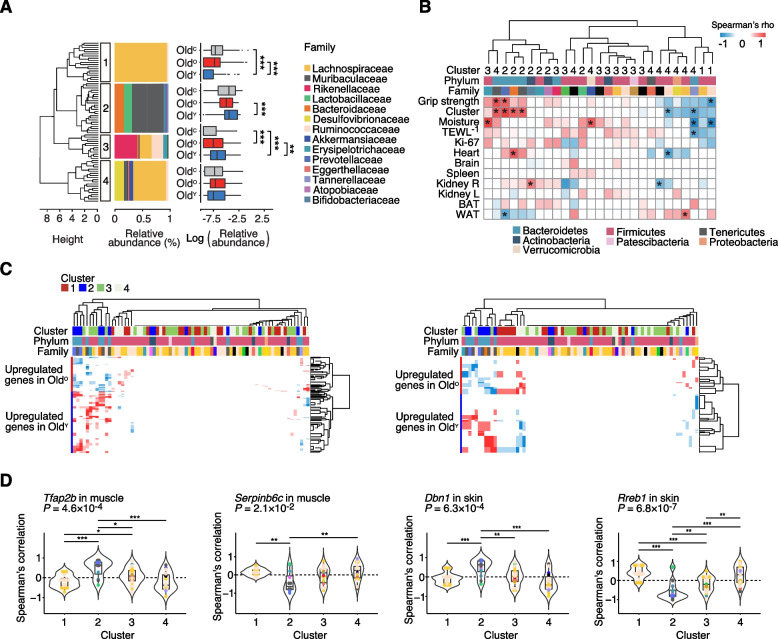
Correlation between co-occurring microbial clusters and host factors. **A** Gut microbiota of Old^C^, Old^O^, and Old^Y^ at week 8 was divided into four clusters by hierarchical clustering analysis with Spearman’s correlation coefficient. The dendrogram was generated by complete hierarchical clustering based on Euclidean distance and four clusters were determined by cutting off the tree at height 6. The taxon bar plot shows the microbial composition of each cluster at the family level, and the distribution of each cluster among the groups was displayed using a boxplot. **B** Heatmap of Spearman’s correlation between bacterial taxa and various phenotypes. **C** Heatmap of Spearman’s correlation between bacterial taxa and DEGs in the muscle (left) or skin (right). Only statistically significant correlations (*P* < 0.05) were plotted. **D** Violin plot representing Spearman’s correlation between microbial clusters and top FCs in the muscle and skin. Colored spots indicate the microbial families and horizontal lines in the box plot show the median. *P* values were calculated using the Kruskal-Wallis test. *P < 0.05, **P < 0.01, ***P < 0.001. Wilcoxon rank sum test.

To define a consortium of core microbial taxa that are involved in aging-associated phenotypes, we clustered the entire families of the gut microbiota at week 8 according to microbial abundance correlation (Fig. 4A). Among the four clusters retrieved by hierarchical clustering, *Lachnospiraceae*-dominant cluster 1 was prevalent in Old^C^ and Old^O^ groups, whereas cluster 2 containing *Muribaculaceae*, *Bacteroidaceae*, *Lactobacillaceae*, and *Prevotellaceae* was abundant in Old^Y^. While cluster 3 that distinctly harbored *Rikenellaceae*, *Ruminococcaceae*, and *Erysipelotrichaceae* was enriched in Old^Y^, it was also significantly enriched in Old^O^ compared to non-FMT control. In addition, *Desulfovibrionaceae*-containing cluster 4 had no preference for any treatments, implying a minor impact of its members on the host (Fig. 4A).

To better understand the association of microbial clusters with host physiology, we calculated Spearman’s correlation coefficient between the cumulative abundance of clusters and the indicators of host fitness (Fig. 4B). *Prevotellaceae* and *Muribaculaceae* in clusters 2 and 4 showed a positive correlation with both grip strength and skin moisture, the most improved phenotypes in Old^Y^. *Bacteroidaceae* in cluster 2 positively correlated with skin moisture and brain size, and *Erysipelotrichaceae* in cluster 3 and *Akkermansiaceae* in cluster 4 with the inverse of TEWL (TEWL^-1^). In contrast, *Ruminococcaceae* in cluster 1 negatively correlated with grip strength and TEWL^-1^, *Tannerellaceae* in cluster 4 with skin moisture, TEWL^-1^, and Ki-67^+^, and *Ruminococcaceae* in cluster 4 with skin moisture and brain size (Fig. 4B). Thus, *Bacteroidetes* families in clusters 2 and 3 are strongly associated with restored phenotypes of the aged hosts, while *Firmicutes* families mainly present in clusters 1 and 4 may promote functional deterioration.

Next, we explored the correlation between tissue-specific DEGs in Old^Y^ and Old^O^ and clustered taxa. *Muribaculaceae*, *Prevotellaceae*, and *Bacteroidaceae* (all in the phylum *Bacteroidetes*) in cluster 2, as well as *Ruminococcaceae* and *Erysipelotrichaceae* in cluster 3, had a strong association with upregulated genes in the muscle and skin of Old^Y^ (Fig. 4C). Conversely, upregulated genes in Old^O^ negatively correlated with cluster 2 families, but positively with *Lachnospiraceae* in clusters 1 and 4 and *Ruminococcaceae* in cluster 1. Similar patterns were observed in the colon tissues (Fig. S4A). Correlation coefficients between the top DEGs and bacterial clusters were compared in each tissue. Cluster 2 showed the highest positive correlation with *Tfap2b* and *Dbn1*, which are most upregulated in the muscle and skin of Old^Y^, respectively. *Serpinb6c* and *Rreb1*, most downregulated in each tissue, respectively, negatively correlated with cluster 2 (Fig. 4D). Similar results were produced for *Trpm3* and *Wdr54* in the colon (Fig. S4B). Consistently, members of *Muribaculaceae*, *Prevotellaceae*, and *Bacteroidaceae* were associated with increased gene expression and improved host phenotypes in Old^Y^, suggesting that they may have an ability to rejuvenate host physiology.

### Dbn1 is involved in water retention in the skin

**Fig. 5:**
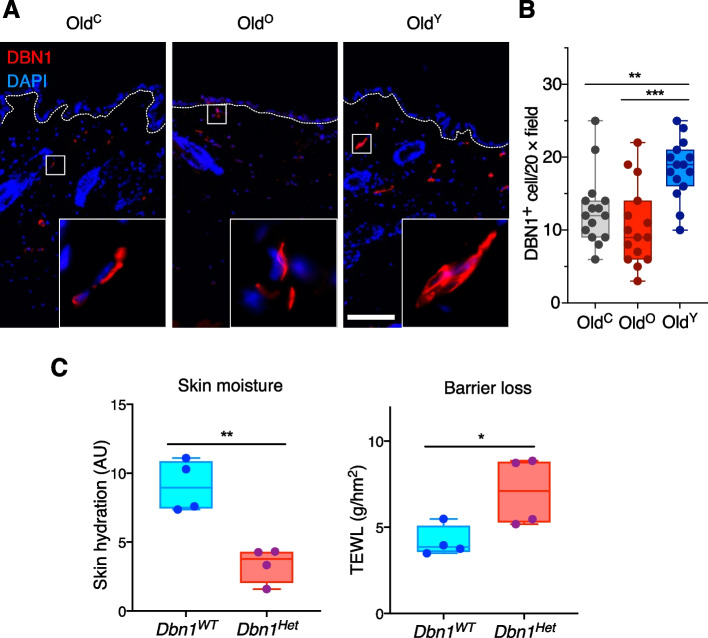
DBN1 is responsible for skin homeostasis. **A** Immunofluorescence staining of DBN1 in the skin of Old^C^, Old^O^, and Old^Y^. **B** Quantification of DBN1^+^ cells in 20 × field. Scale bar = 100 μm. Box and whiskers. One-way ANOVA. **C** Skin hydration (left) and TEWL (right) values in *Dbn1*^*WT*^ and *Dbn1*^*Het*^ mice. Box and whiskers. Student’s *t* test. *P < 0.05, **P < 0.01, and ***P < 0.001

Notably, among the sets of DEGs, *Dbn1* in the skin of Old^Y^ was most upregulated across the three tissues (171.1 fold increase; *P* = 0.001) (Table S3). Thus, we examined the in situ expression of DBN1 via immunofluorescence and found that the dermis of Old^Y^ harbored about two times more DBN1-positive (DBN1^+^) cells than those of Old^C^ and Old^O^ (Fig. 5A, B). To validate the functionality of *Dbn1*, we measured the water retention capacity of the wild type and *Dbn1*-heterozygous (*Dbn1*^*Het*^) C57BL/6 littermates (8 weeks old). While the levels of skin hydration and TEWL were comparable between young *Dbn1*^*WT*^ and Old^Y^, the halved gene dosage of *Dbn1* resulted in impairment of skin hydration and barrier function as much as those observed in Old^O^ (Fig. 5C). These data indicate that the gut microbiota derived from the young improves water-holding capacity in perimenopause female mice by modulating host gene expression like upregulation of *Dbn1*.

## Discussion

Perspectives on understanding aging are important for intervention and management of the process. The concept of “programmed aging” that considers aging as a designed biological program allows, at least partially, the rejuvenation of old body through the modulation of effector(s) [33]. Since Smith and colleagues first reported extension of life span and delayed behavioral decline in short-lived killifish by the young-derived microbiota [19], a few groups have added evidence on the counteracting effects of fecal microbiota transfer on hallmarks of aging gut, brain, and retina using the mouse model [34–36]. Our results highlight that aging phenotypes of physical fitness in aged mice can be ameliorated by transplanting the young-derived microbiota.

Indicators of muscle and skin properties including forelimb grip strength, the thickness of muscle fiber, number of stratum corneum layers, skin water retention, and skin cell markers (Ki-67 and KRT10) were all improved in old mice at 12 months with or without Abx pre-treatment. Further, a statistically significant increase in muscle fiber and brain size was observed in very old mice at 25 months. A couple of studies reported that gut microbiota is implicated in the skeletal muscle function. Germ-free mice suffer from reduced muscle mass and locomotor activity as compared to mice raised under the SPF condition [27], and xenogeneic transplantation of the pig gut microbiota to germ-free mice synchronized lipid metabolisms in muscle, collectively supporting the effect of the gut microbiota in the muscle [27, 37].

Among all organs measured, only the brain, usually weighing 2% of the body weight, showed a moderate change by fecal microbiota transfer. Old mice at 12 months receiving the gut microbiota from the young maintained their relative brain mass regardless of Abx pre-treatment, but those receiving the old-derived microbiota showed a slight decrease. Despite the change, avoidance-learning memory was not different among them, implying that the behavioral relevance of the reduced brain size in xOld^O^ is trivial. Of note, there was a significant increase in brain size for vOld^Y^ as compared to vOld^VO^ and vOld^C^; however, the physical activity of the very old mice was too low for the passive avoidance test as well as the grip strength test. Given the pre-documented association between the gut microbiota and brain function [35, 36], these results may serve as preliminary data for a further study on the physical and functional fitness of the aging brain. The two studies revealed the beneficial effects of the young microbiota, altering the immune profiles in the gut and brain. The results are in line with the “inflammaging”| theory; however, it is hard to distinguish the true anti-inflammatory activity of the young-derived microbiota from the absence of the old-derived immunogenic molecules. Transplantation of the fecal microbiota from old mice did not induce significant changes in body weight, organ mass, the level of biomarkers for tissue damage, pro-inflammatory cytokines, and the number of T cells in our analyses (data not shown), suggesting that ours are not mediated by the lack of the old-derived immunogenicity.

We identified that a group of microorganisms consisting of *Muribaculaceae*, *Prevotellaceae*, and *Bacteroidaceae* (all *Bacteroidetes*) represents the young-derived microbiota that are discriminative in old mice at 12 months after fecal transfer and strongly correlates with enhanced gene expression of the recipients’ muscle and skin. This is in line with our big data analysis of the human gut microbiota with conserved enrichment of *Bacteroidaceae* in the young population (unpublished data) and a previous report on high frailty of old people having reduced *Bacteroides* and *Prevotella* [13]. Moreover, these three families co-occurring in a *Bacteroidetes*-rich cluster based on hierarchical clustering are significantly associated with higher grip strength and improved skin water retention. Microbial taxa more abundant in aged mice and likely to contribute to aging phenotypes were also defined. Some species of *Lachnospiraceae* and *Ruminococcaceae* in the phylum *Firmicutes* were consistently over-represented in old mice that received the old-derived microbiota, and correlated with upregulated genes in the muscle and skin. In addition, a few members of *Ruminococcaceae* are negatively associated with grip strength, skin water retention, and brain size. Our results provide a list of causative bacteria that may induce host gene expression and subsequent phenotypic changes with regards to revitalizing the aged.

Finally, we elucidated that *Dbn1*, highly induced in the skin of Old^Y^ mice, exerts a protective role in skin hydration. Although DBN1, a cytoplasmic actin-binding protein, is known to be expressed ubiquitously, its function has been documented mainly in neurobiology such as neuronal stem cell differentiation and cognitive function in Alzheimer’s disease [38, 39]. Our results are the first evidence that relates *Dbn1* to skin hydration; however, considering the predefined role of *Dbn1*, it is plausible that DBN1 may facilitate the proliferation or differentiation of epidermal stem cells, leading to the improved skin phenotypes, which awaits further investigation.

## Conclusions

The interaction between the gut microbiome and the host has been shown to modulate host physiology, which inspired people to examine the revitalizing potential of the gut microbiota from the young. While impaired physical fitness such as sarcopenia and skin barrier dysfunction in older people has corroborated the “damage theory” of aging, our study for the first time demonstrates that microbial intervention strengthens the aged muscle and rejuvenates the skin through changes in the microbial structure and host gene expression. We proposed *Dbn1* as an important player in maintaining skin integrity and further identified microbial members related to pro- or anti-aging phenotypes. Our study provides comprehensive evidence of the revitalization of multiple organs in the host and points to host and microbial targets for anti-aging treatment.

## Supplementary Information

**Additional file 1: Figure S1.** Physiological characteristics of old mice exposed to the three treatments. **Figure S2.** Physiological characteristics of very old mice transplanted with microbiota from control, very old, and young mice. **Figure S3.** Immune profile of FMT mice. **Figure S4.** Correlation analysis between microbial clusters and DEGs in the colon tissue.

**Additional file 2: Table S1.** List of genes corresponding to Fig. 3B. **Table S2.** List of genes corresponding to Fig. 3E. **Table S3.** List of genes corresponding to Fig. 3H.

## Data Availability

The sequencing reads of the 16S rRNA gene for the mouse fecal microbiota have been deposited in GenBank under the BioProject number PRJNA789433 (https://dataview.ncbi.nlm.nih.gov/object/PRJNA789433?reviewer=p6bokr5gnifd4atmgjj9f6dik2).

## Abbreviations

Abx: Antibiotic cocktail
ALB: Albumin
ALP: Alkaline phosphatase
AMYL: Amylase
AU: Arbitrary unit
ASV: Amplicon sequence variant
BUN: Blood urea nitrogen
CPK: Creatinine phosphokinase
CRE: Creatinine
DEG: Differentially expressed gene
ERG: Electronretinogram
FMT: Fecal microbiota transplantation
GGT: R-glutamyltransferase
CKMB: Creatine kinase-mb
GOT: Aspartate aminotransferase
GPT: Alanine aminotransferase
GSEA: Gene set enrichment analysis
HDLC: High-density lipoprotein-cholesterol
H&E: Hematoxylin and eosin
IOP: Intraocular pressure
KEGG: Kyoto Encyclopedia of Genes and Genomes
LDA: Linear discriminant analysis
LDH: Lactate dehydrogenase
LEfSe: Linear discriminant analysis effect size
OKN: Optokinetic nystagmus
OTU: Operational taxonomic unit
PCoA: Principal coordinate analysis
TBIL: Total bilirubin
TCHO: Total cholesterol
TEWL: Transepidermal water loss
TG: Triglyceride
