# Supplementary material for: Gut microbiota of the young ameliorates physical fitness of the aged in mice
Source: Microbiome. 2022 Dec 26;10:238. doi: 10.1186/s40168-022-01386-w (PMC9791737; doi:10.1186/s40168-022-01386-w)
Supplement: Supplementary file 2 — Additional file 1: Figure S1. Physiological characteristics of old mice exposed to the three treatments. Figure S2. Physiological characteristics of very old mice transplanted with microbiota from control, very old, and young mice. Figure S3. Immune profile of FMT mice. Figure S4. Correlation analysis between microbial clusters and DEGs in the colon tissue. [file 40168_2022_1386_MOESM1_ESM.docx]

**Supplementary information for**

**Gut microbiota of the young ameliorates physical fitness of the aged in mice**

**Kwang H. Kim, Yusook Chung, Ji-Won Huh, Dong Jin Park, Yejin Cho, Yeseul Oh, Haengdueng Jeong**, **Jaekyung Yoon, Ju-Hee Kang, Hae-Sol Shin, Hyoung-Chin Kim, Soon-Kyeong Kwon, Kyoung Yul Seo, Seung Hyun Oh, Je Kyung Seong, Sang-Jun Ha, Ki Taek Nam and Jihyun F. Kim**

E-mail addresses: ucman0724@yuhs.ac; yusookzzz@yonsei.ac.kr; jwhuh@yonsei.ac.kr; djpark11@gmail.com; MAMAYJ87@yuhs.ac; ohyes322@yuhs.ac; pwrttony@yuhs.ac; ymicrobes@yonsei.ac.kr; applekjh0503@hanmail.net; HSOL916@yuhs.ac; hckim@kribb.re.kr; skkwon@gnu.ac.kr; SEOKY@yuhs.ac; eyeball@gachon.ac.kr; snumouse@snu.ac.kr; sjha@yonsei.ac.kr; kitaek@yuhs.ac; jfk1@yonsei.ac.kr

**This PDF file includes:**

Figures S1–S4


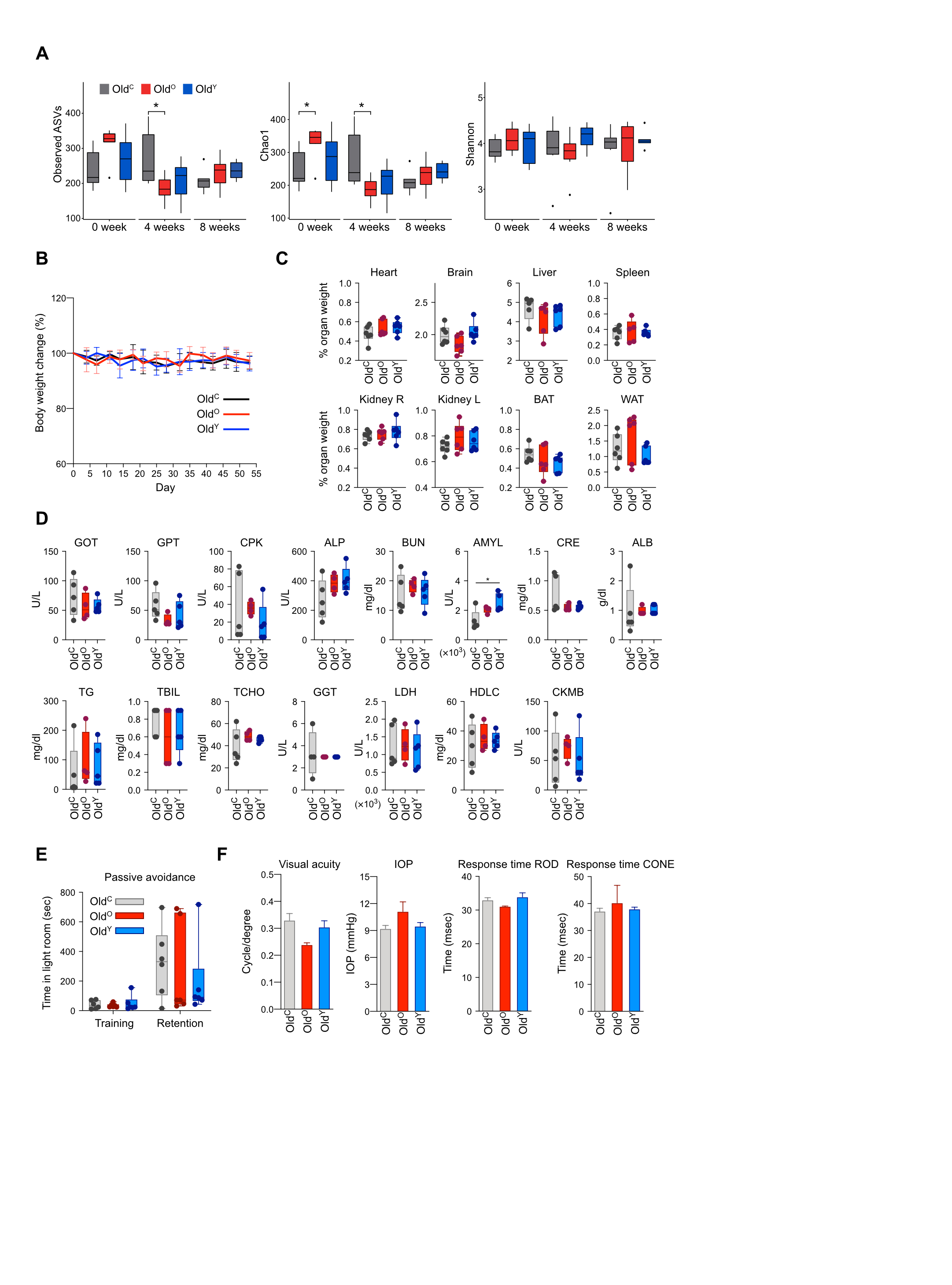


**Fig. S1. Physiological characteristics of old mice exposed to the three treatments. A** Alpha diversity profile of the recipient mice during FMT. Wilcoxon rank-sum test was applied. **B** Body weight change during FMT. Old^C^, Old^O^, and Old^Y^ groups of mice were weighed twice per week during FMT, and housed in specific pathogen-free facility (n = 6). Results are shown as mean ± SD. **C** Relative organ weight of old FMT mice. The heart, brain, liver, spleen, kidney, brown adipose tissue (BAT), and white adipose tissue (WAT) were weighed three days after the last FMT. **D** Blood chemistry analysis of serum samples from the treatment groups. GOT, aspartate aminotransferase; GPT, alanine aminotransferase; CPK, creatinine phosphokinase; ALP, alkaline phosphatase; BUN, blood urea nitrogen; AMYL, amylase; CRE, creatinine; ALB, albumin; TG, triglyceride; TBIL, total bilirubin; TCHO, total cholesterol; GGT, γ-glutamyltransferase; LDH, lactate dehydrogenase; HDLC, high density lipoprotein-cholesterol; CKMB, creatine kinase-MB. **E** Passive avoidance test. Total time in the illuminated chamber was measured at day 1 (training time) and retention time was measured at day 2. **F** Visual activity measurement. Visual acuity, intraocular pressure (IOP), and electroretinogram (ERG) analysis were performed. **P* < 0.05, ***P* < 0.01.

**
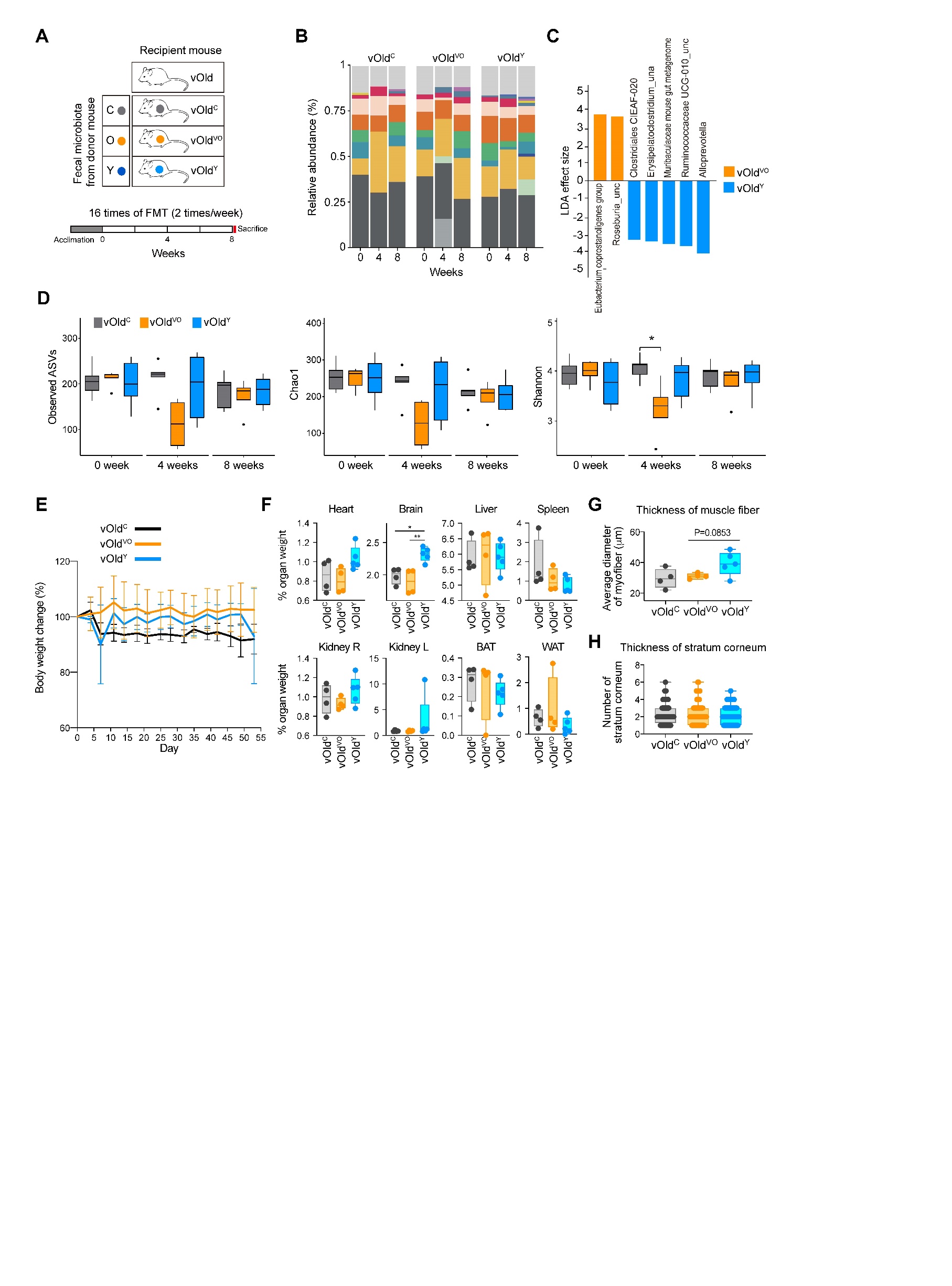
**

**Fig. S2. Physiological characteristics of very old mice transplanted with microbiota from control, very old, and young mice.** **A** A schematic illustration of FMT of 25-month-old mice (very old mice). The matrix describes three donor groups: C, PBS with 10% glycerol for control; O, very old at 25 months; Y, young at 5 weeks. The recipient mice are designated vOld^C^ (n = 6), vOld^VO^ (n = 4) and vOld^Y^ (n = 5) respectively. After 2 weeks acclimation period, FMT was repeated 16 times for 8 weeks. **B** Relative abundance of the gut microbiota of the recipient mice during 8 weeks of FMT. Samples are grouped by fecal donor and ordered by collection time. Color code for taxonomy is identical to figure 1A. **C** Linear discriminant analysis effect size of samples between vOld^VO^ and vOld^Y^. _una, unassigned; _unc, uncultured. **D** Alpha diversity profile of the recipient mice during FMT. Wilcoxon rank-sum test was applied. **E-H** Determination of physiological changes in very old FMT mice. vOld^C^, vOld^VO^, and vOld^Y^ groups of mice were housed in a specific pathogen-free facility and weighed twice per week during FMT. Results are represented as mean ± SD (**E**). The heart, brain, liver, spleen, kidney, BAT, and WAT were weighed three days after the last FMT (**F**). Average diameter of muscle fiber in each group was determined (**G**). Average number of stratum corneum layers was counted (**H**). **P* < 0.05, ***P* < 0.01.


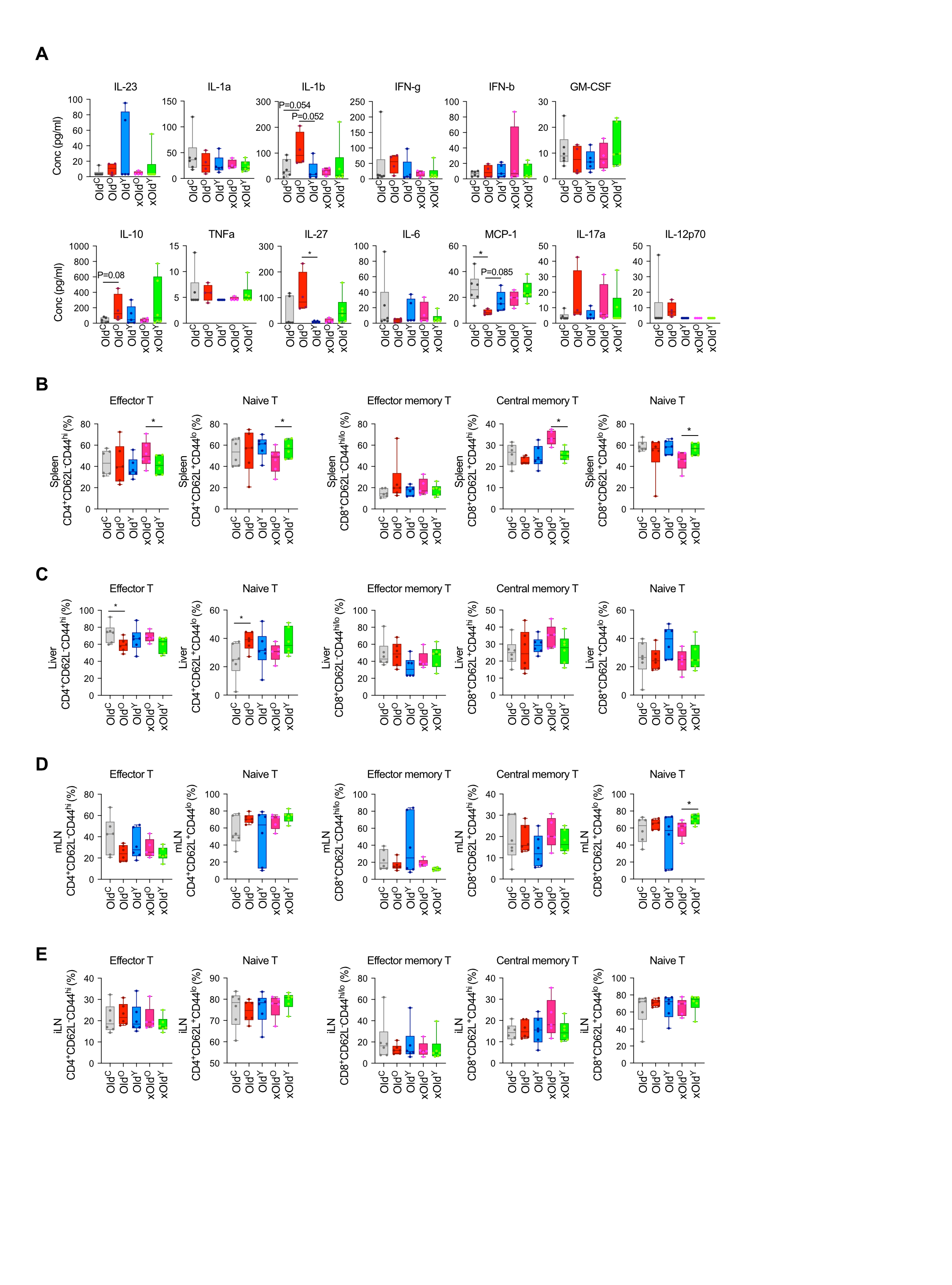


**Fig. S3. Immune profile of FMT mice**. **A** Serum cytokine level of FMT mice. A total of 13 cytokines were detected using LEGENDplex Mouse Inflammation panel. Statistical analysis was conducted using unpaired two-tailed *t*-test. **B-E** T cell profile of FMT mice. Representative flow cytometry plots and frequencies of indicated subsets of CD4 and CD8 T cells in spleen (**B**), liver (**C**), mLN (**D**), and iLN (**E**). One-way ANOVA. * *P* < 0.05.


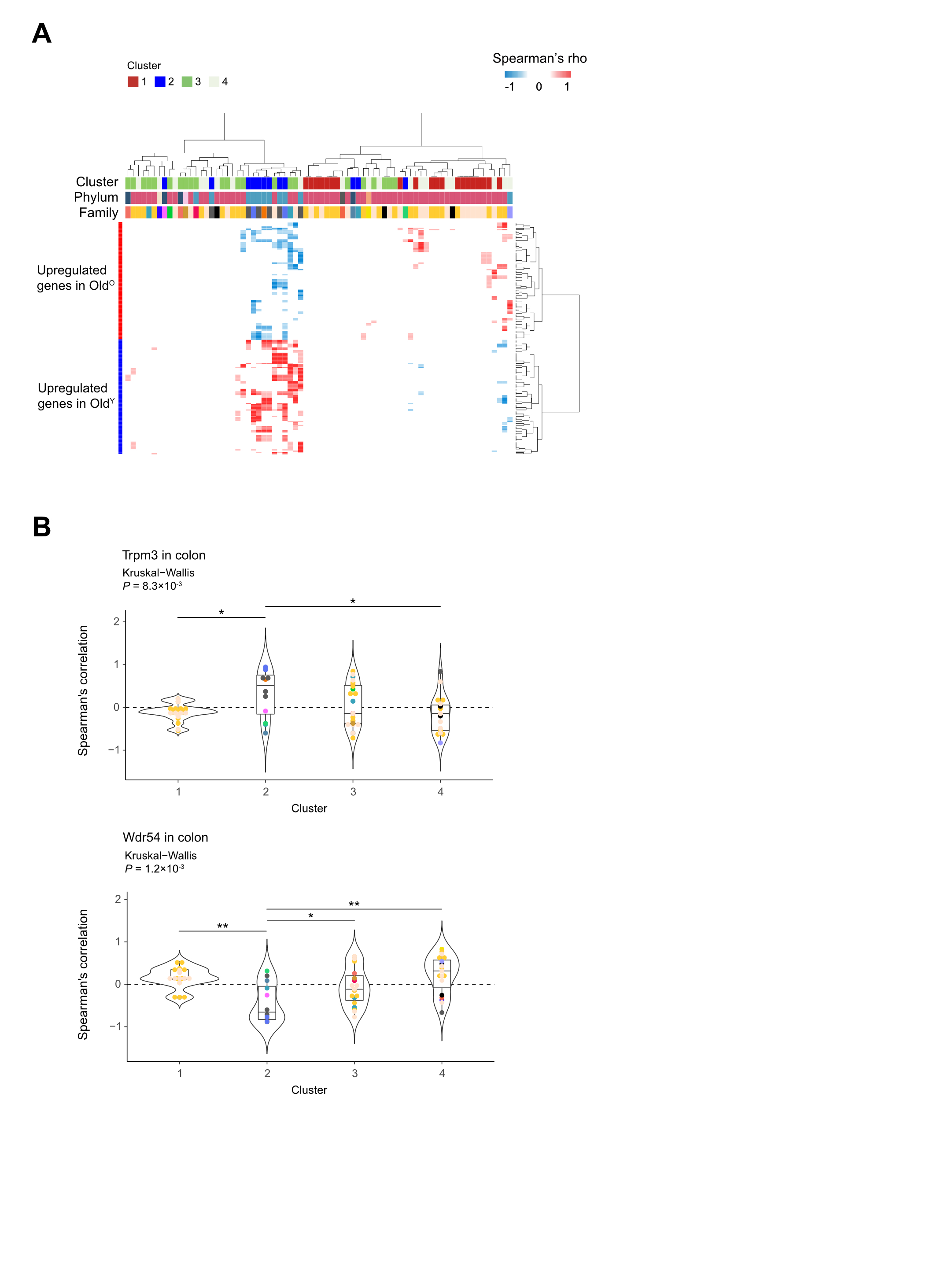


**Fig. S4. Correlation analysis between microbial clusters and DEGs in the colon tissue. A** heatmap of Spearman’s correlation between taxa and significant DEGs in colon (left). Only statistically significant correlations were plotted (*P* < 0.05). Color code for taxonomy is identical to figure 1A. **B** Violin plot representing Spearman’s correlation between microbial clusters and top significant DEGs in colon. Colored spot indicates the microbial family and horizontal line in box plot shows median correlation coefficient. *P*-values were calculated using the Kruskal-Wallis test. **P* < 0.05, ***P* < 0.01, ****P* < 0.001.
